# In this issue: Volume 115, Issue 7, July 2024

**DOI:** 10.1111/cas.16257

**Published:** 2024-07-12

## Clonal expansion in normal tissues

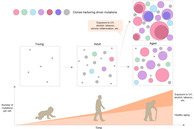

Cancer starts from a single cell that begins to grow and divide uncontrollably, forming a tumor. This often happens due to genetic mutations, but since many mutations appear in later‐stage cancers, finding the exact cause can be difficult. Recent studies reveal that these mutations can accumulate in normal tissues as we age. Factors like alcohol, smoking, and UV rays accelerate this process.

In this review article, Maeda and Kakiuchi shed light on the accumulation of these mutations and explore how they might lead to cancer. They describe that our tissues tend to accumulate mutations as we age, even if we are healthy. Studies on blood cancer and certain breast cancers show that sometimes, these mutations occur very early in life and remain inactive for decades until cancer eventually develops. Interestingly, these mutated cells in blood, known as clonal hematopoiesis (CH), are also linked to other diseases, such as heart disease and severe COVID‐19.

Another factor that influences the risk of cancer development is the order and timing of these mutations. Cells face different demands and “selective pressures” at different life stages. A mutation that might give a cell an advantage early on could become less beneficial or even detrimental later. The specific order in which mutations appear also influences how they interact with each other and can determine the cell's fate.

This research highlights the double‐edged nature of these mutated cells. While they can lead to cancer, they also hold clues to understanding and treating many diseases. Using advanced sequencing tools, scientists can analyze cancer genomes and identify many genes responsible for cancer. This technology additionally assists in monitoring the development of cancer over time by revealing the sequence in which mutations occur. However, understanding the early steps of cancer development is still difficult.

Overall, the researchers emphasize that cancer development is a complex process influenced by not just the presence of mutations, but also the specific context in which they occur.

https://onlinelibrary.wiley.com/doi/10.1111/cas.16183

## In‐depth immune profiling of peripheral blood mononuclear cells in patients with pancreatic ductal adenocarcinoma reveals discriminative immune subpopulations

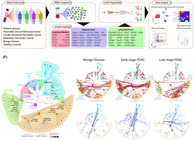

Pancreatic ductal adenocarcinoma (PDAC) is a highly aggressive form of pancreatic cancer with a 5‐year survival rate of less than 10%. One primary reason for the high mortality rates in PDAC is delayed diagnosis, which limits the effectiveness of surgery and chemotherapy.

In this study, Rodriguez et al. conducted an in‐depth analysis of the immune profile of patients with PDAC. The findings could aid in the development of novel treatment strategies and assist in the early detection of PDAC.

The researchers divided the study participants into four groups: patients with resectable PDAC (where the tumor could be removed by surgery), patients with irresectable PDAC (where surgical removal of the tumor was not possible), patients with benign tumor controls (non‐cancerous diseases of the pancreas), and healthy controls. They then performed flow cytometry analysis on blood samples from these patients.

The study found that PDAC patients exhibited a complex immune profile, showing both anti‐ and pro‐tumor immune responses. Both PDAC and benign patients showed an increase in CD86^+^ classical monocytes and memory T cells expressing CCR6 and CXCR3, which are associated with T‐helper 1 (Th1) and Th17 immune responses, respectively. These responses may suggest better survival rates.

However, PDAC patients also showed an increase of CD39^+^ regulatory T cells and CCR4^+^CCR6^−^CXCR3^−^ T cells, indicating the presence of Th2‐like immune responses. Th2‐associated cytokines are known to be associated with poor survival. PDAC patients showed a unique immune profile with both supportive and suppressive elements, compared to other groups.

Further studies with larger patient groups are needed to better understand the correlation between immune cells and their clinical impact on PDAC.

https://onlinelibrary.wiley.com/doi/10.1111/cas.16147

## Fat and proteolysis due to methionine, tryptophan, and niacin deficiency leads to alterations in gut microbiota and immune modulation in inflammatory bowel disease

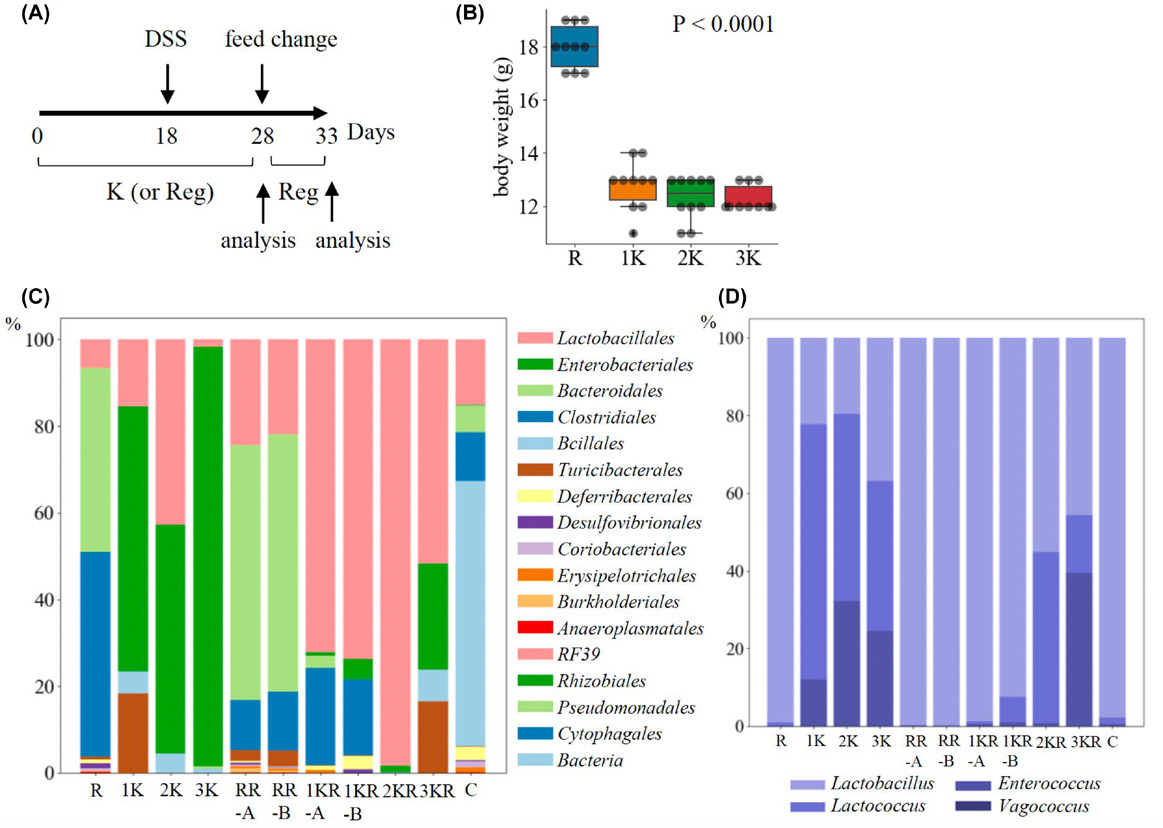

Inflammatory bowel disease (IBD) is a chronic inflammatory disorder of the gastrointestinal tract with symptoms ranging from severe abdominal pain to debilitating diarrhea. Previous studies have highlighted the role of the nutrients methionine, tryptophan, and niacin (MTN) in the context of IBD. However, the precise implications of various nutrient deficiencies on gut health are not well understood.

Against this backdrop, Hara et al. conducted a study to elucidate the effects of MTN deficiencies in mouse models of IBD, providing insights into their potential contribution to disease progression.

The study employed dextran sodium sulfate (DSS)‐induced colitis mice that were divided into groups and fed diets deficient in methionine, niacin and tryptophan, or all three. Fecal and colonic samples were collected for analysis, and gut microbiota were assessed using 16S rRNA sequencing.

The results revealed significant alterations in gut microbiota composition and host gene expression due to MTN deficiencies. Mice fed MTN‐deficient diets exhibited changes in the abundance of specific bacterial taxa, such as a decrease in *Lactobacillus*, which is responsible for suppressing inflammation and regulating immune responses. Furthermore, modulation of immune‐related cytokines was observed, with both pro‐inflammatory and anti‐inflammatory cytokines showing altered expression patterns. Notably, the expression of genes involved in fat and protein degradation also increased due to MTN deficiency, which led to weight loss in mice. The study also looked into the effects of switching from MTN‐deficient to normal feed and discovered that the percentage of *Lactobacillus* increased when returning to the regular diet.

In conclusion, MTN deficiencies significantly alter gut microbiota composition and host gene expression in mouse models of IBD. This study underscores the potential of dietary interventions targeting MTN deficiencies for IBD prevention and treatment. It also highlights the importance of MTN metabolism for maintaining gut health and immune function.

https://onlinelibrary.wiley.com/doi/10.1111/cas.16153

